# Amphetamine Sensitization Alters Reward Processing in the Human Striatum and Amygdala

**DOI:** 10.1371/journal.pone.0093955

**Published:** 2014-04-09

**Authors:** Owen G. O’Daly, Daniel Joyce, Derek K. Tracy, Adnan Azim, Klaas E. Stephan, Robin M. Murray, Sukhwinder S. Shergill

**Affiliations:** 1 Cognition, Schizophrenia & Imaging Laboratory, Department of Psychosis Studies, the Institute of Psychiatry, King’s College London, London, United Kingdom; 2 Department of Neuroimaging, Centre for Neuroimaging Sciences, the Institute of Psychiatry, King’s College London, London, United Kingdom; 3 Oxleas NHS Foundation Trust, London, United Kingdom; 4 Laboratory for Social and Neural Systems Research, Department of Economics, University of Zürich, Zürich, Switzerland; 5 Wellcome Trust Centre for Neuroimaging, Institute of Neurology, University College London, London, United Kingdom; 6 Department of Psychosis Studies, the Institute of Psychiatry, King’s College London, London, United Kingdom; 7 The National Psychosis Unit, South London, and Maudsley NHS Foundation Trust, London, United Kingdom; Duke University Medical Center, United States of America

## Abstract

Dysregulation of mesolimbic dopamine transmission is implicated in a number of psychiatric illnesses characterised by disruption of reward processing and goal-directed behaviour, including schizophrenia, drug addiction and impulse control disorders associated with chronic use of dopamine agonists. Amphetamine sensitization (AS) has been proposed to model the development of this aberrant dopamine signalling and the subsequent dysregulation of incentive motivational processes. However, in humans the effects of AS on the dopamine-sensitive neural circuitry associated with reward processing remains unclear. Here we describe the effects of acute amphetamine administration, following a sensitising dosage regime, on blood oxygen level dependent (BOLD) signal in dopaminoceptive brain regions during a rewarded gambling task performed by healthy volunteers. Using a randomised, double-blind, parallel-groups design, we found clear evidence for sensitization to the subjective effects of the drug, while rewarded reaction times were unchanged. Repeated amphetamine exposure was associated with reduced dorsal striatal BOLD signal during decision making, but enhanced ventromedial caudate activity during reward anticipation. The amygdala BOLD response to reward outcomes was blunted following repeated amphetamine exposure. Positive correlations between subjective sensitization and changes in anticipation- and outcome-related BOLD signal were seen for the caudate nucleus and amygdala, respectively. These data show for the first time in humans that AS changes the functional impact of acute stimulant exposure on the processing of reward-related information within dopaminoceptive regions. Our findings accord with pathophysiological models which implicate aberrant dopaminergic modulation of striatal and amygdala activity in psychosis and drug-related compulsive disorders.

## Introduction

Repeated intermittent administration of psychostimulants, such as cocaine or amphetamine, is associated with a progressive sensitivity to the drug’s effects [1]–[3], termed sensitization. In rodent models drug-induced hyperlocomotion and increased sensitivity to stressors are commonly observed [4], [5], associated with an enhanced ability of the drug [6], [7], or a stressor [8], to release dopamine (DA) in the nucleus accumbens. Such dysregulation of mesolimbic DA signalling has been posited as a model of dopaminergic abnormalities during the development of schizophrenia [9], [10], drug addiction [11], their co-morbidity [12], drug-induced psychosis [13] and impulse control disorders seen in some patients with Parkinson’s disease following chronic exposure to dopamine agonists [14], [15]. Support for sensitization in these disorders comes from PET studies showing enhanced striatal DA release in response to dopaminergic agonists in patients with schizophrenia [16]–[18], and in those with Parkinson’s disease showing compulsive drug-seeking behaviour [19] or pathological gambling [20] after chronic DA agonist use. However whilst a large amount of rodent data support a role for DA sensitization in the development of drug-self-administration and drug-seeking behaviour [21]–[23], decisive PET data in human drug dependency is relatively lacking [24]–[26], though enhanced dorsal striatal DA release in response to drug-related cues has been reported [27], [28].

The midbrain dopaminergic nuclei lie at the heart of the brain’s reward circuitry, projecting to targets that include the striatum, nucleus accumbens, the orbitofrontal cortex and the amygdala [29]–[31]. Contemporary theories suggest that phasic DA release provides a signal of any discrepancy between received and anticipated reward (i.e. reward prediction error) which is a vital “teaching signal” for learning [32]–[34]. Individuals with schizophrenia have been demonstrated to show abnormal associative learning [35]–[37] and reward-related BOLD signalling [38]–[40], consistent with disruption of these processes being part of the pathophysiology of this illness [41]. Interestingly, drug addiction is associated with deficits on tasks linked to orbitofrontal cortical function [42]–[45] and aberrant reward prediction error signals [46]–[48]. However, dopamine signalling has also been implicated in the attribution of motivational significance, or incentive salience, to environmental cues. In fact, one contemporary theory of drug addiction suggests an augmentation of this processes, as a results from mesolimbic sensitization following repeated drug exposure, explains the powerful motivation for drugs for addicts [49], [50]. This incentive sensitization mechanism has also recently been used to explain the phenomenology of schizophrenia, with patients reporting that the world seems imbued with personal significance [51]. The rewarded gambling task, which includes a rewarded outcome and anticipation conditions may permit us to explore the effects of amphetamine sensitization with respect to both of these models.

Sensitised rodents display similar reward-related deficits [52]–[55], aberrant learning [56], [57], and abnormal striatal and orbitofrontal activity [58]. While there is considerable evidence for the development of AS in primates [59]–[61], to date no formal examination of reward processing in humans has taken place. Preliminary behavioural data suggested that sensitization could be safely induced in healthy human subjects [62]–[64]: recent PET studies have explored the effects of a sensitising dosage regime of amphetamine on drug-induced dopamine release [65], the contribution of conditioning to this effect [66] and, more recently, its interaction with stress [67]. In healthy male volunteers, these data demonstrated that AS was associated with enhanced drug-induced dopamine release in the ventral striatum, extending dorsally into the dorsal caudate and putamen [65].

In this present study we used the same amphetamine dosage regime as Boileau et al and employed a rewarded gambling (wheel-of fortune) task [68], [69] to explore the impact of sensitization on the ability of a low dose of amphetamine to modulate different aspects of reward processing - namely decision making, anticipation and outcome processing - in dopaminoceptive brain regions. We hoped to show, for the first time, that sensitization would induce altered BOLD signal in key regions of reward-processing circuitry, specifically the striatum, the orbitofrontal cortex and the amygdala, during the various phases of our gambling task, although given the incentive sensitization hypothesis, we propose that reward anticipation is of most interest in this regard.

## Methods

### Participants and Design

Our study was designed to explore (1) the feasibility of demonstrating the translation of a rodent model of dopamine dysregulation to humans and (2) to characterise the neural substrates of such mesolimbic sensitization on reward processing in humans. We were not exploring the effects of repeated intermittent stimulant exposure on any clinically relevant measures, nor were we exploring the efficacy of this procedure using any standard randomised clinical trials design. As such this work was not deemed a clinical trial by the United Kingdom MHRA or local ethics committee. However, full ethical approval for this research project was received from the King’s College London’s Institute of Psychiatry, Research Ethics Committee reference# 022/03). Participants were provided with information sheet at least 24 hours prior to giving consent to take part in the study. They were given the opportunity to ask questions prior to giving written informed consent. The wording of both the participant information sheet and the consent form were approved by the local ethics committee.

The data reported here is from the same participants as in our previous paper [70] but salient details will be repeated here in brief. Twenty-two right handed male volunteers (age 30.8 years +/−8.5 years), were recruited and assigned to receive either four oral doses of dexamphetamine (20mg), or four doses of a placebo, following a procedure (albeit with a fixed dose across all participants) previously shown to produce dopaminergic sensitization in humans [65]. Subjects received the first 3 doses with a 48-hour inter-dose interval (Sessions 1–3) and again (4th dose) after a two week wash-out period (Session 4) using a double-blind procedure. Participants were excluded if they had any past medical history of note, were taking any medications, or had a family history of mental illness or substance abuse problems, these factors were assessed by a clinician and contact with the participants’ general medical practitioner. However, no standardised psychiatric interview was carried out. Each visit had an initial drug-urine analysis to exclude the use of recreational drugs. The subjects in both the placebo and amphetamine groups were matched in terms of age (p<0.688) and years of education (p<0.99). Drug use was assessed with a set of five–point scales. Subjects were asked “Have you used any of these drugs in the past?” and responded zero for no previous use, one for experimental use (has tried sporadically), two for occasional use (uses small quantities from time to time), three for moderate use (small quantities regularly/large amounts occasionally), and four for severe use (frequent use of large quantities, often to intoxication/debilitation). Subjects scoring three or four were excluded. Neither group differed significantly on these scales for marijuana (Mean (SD); Placebo 1.25(1.1); Amphetamine 0.636(0.673); p<0.122) or other drug use (Mean(SD); Placebo 0.54(0.52); Amphetamine 0.26(0.47); p<0.212). Participants were also excluded if they were proficient in playing a musical instrument or touch typing due to the inclusion of a motor sequence learning task [71] in the scanning battery. During screening all subjects were exposed to a mock-scanner to acclimatise them to the scanner environment, and thereby reduce session differences related to the novelty of the scanning environment.

Note that the context in which the drug was administered, and where participants waited prior to the scan, was carefully controlled, with the same room employed for all visits. Participants were scanned approximately 120 minutes post-drug/placebo administration during sessions 1 (acute exposure) and 4 (following repeated exposure) in an effort to model the effects of sensitization-related dopaminergic dysregulation on the neural substrates of explicit motor sequence learning. During the same scanning session participants also performed a working memory task, a motor learning task and a rewarded gambling task: the findings related to the first two of these tasks are reported elsewhere.

### Acquisition of Functional MRI Data

Imaging was performed with a 1.5T GE scanner (GE, USA). 180 volumes (matrix size 64_×_64) with whole brain coverage were acquired during each functional run. Each volume comprised 36 slices, collected in an interleaved manner, with a slice thickness of 3mm and a 0.3mm gap between slices. The repetition time was 4 seconds, TE = 40ms, flip angle = 90°. Total acquisition time was 18 minutes (1080 seconds). High resolution structural scans were also acquired (Spoiled Gradient Recalled (SPGR) and High-Resolution Gradient Echo).

### Rewarded Gambling Task

**Figure 1:**
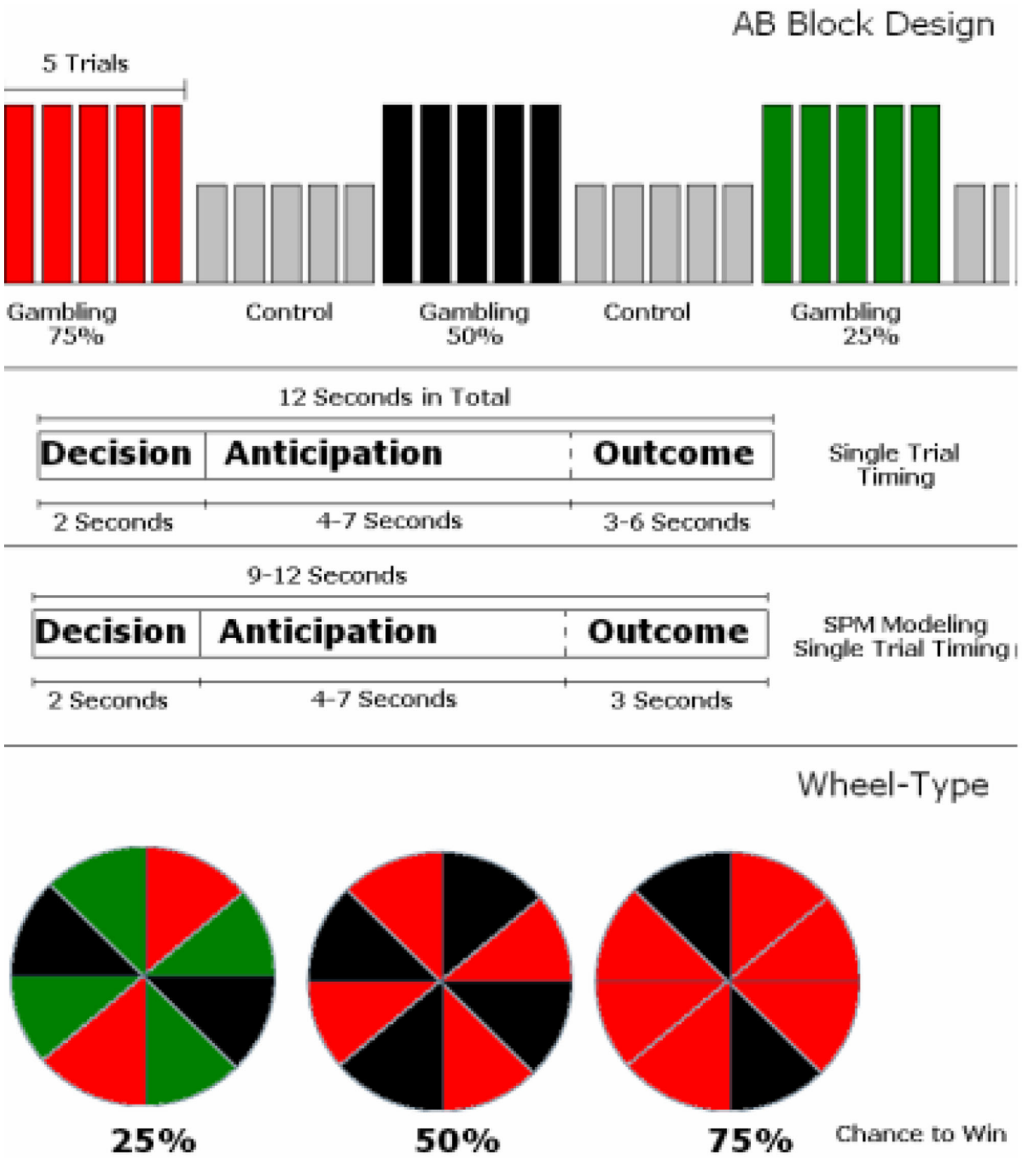
Experimental Design of the Rewarded Gambling Task. (Top) The task had a blocked design where gambling blocks were interleaved with control blocks. Each block consisted of 5 trials, each of 12 seconds length. (Middle) Each trial was 12 seconds long and included a 2 second decision period where a wheel (see bottom) was presented which indicated the perceived probability of winning on this trial. During gambling trials participants were required to make a choice (Red or Black) or they would simply lose the compulsory £1 wager automatically. In the control condition, subject were still asked to make a choice but were limited to colours other than Red and Black and no money was wagered. Following the decision, a variable anticipatory delay (4–7 seconds) preceded a 3–6 second outcome phase where the participant was informed of the outcome of the trial and their balance was amended. Three wheel types were available, each indicating the probability of winning the participant should expect on that trial.

Whilst lying in the scanner subjects performed a block-design rewarded gambling task (Figure 1). Participants were given a starting balance of £15 and informed that they would receive any end balance on completion of the task. On each trial, each participant was presented with a roulette wheel with 8 sectors. Three wheel types were employed, with the proportion of red and black sectors on the wheel manipulated to set the perceived likelihood of winning at 25%, 50%, and 75%. In fact, the outcomes were fixed on a trial-by-trial basis to ensure an approximately equal number of wins and loss trials over the session. In the decision phase of the task, participants were presented a cue indicating whether this was a gambling (wager £1) or control (wager £0) trial and prompting them to make a choice (red or black when gambling, or yellow and blue during control trials) within a 2 second decision window: failure to make a choice led to an automatic loss of £1 on the gambling task. The task involved alternating 36 second blocks of 5 trials (either rewarded or control). This reward-control block cycle was repeated 3 times, giving a total of 15 trials for each probability wheel (25%, 50%, and 75%). Following the 2 second decision phase, the subjects waited for a variable delay period of between three and seven seconds (anticipation) during which the roulette ball rotated around the outside of the roulette wheel. When the ball came to rest subjects were informed of the outcome (win or loss) and their money total was amended accordingly (+£1 for a win, -£1 for a loss). The total trial length was fixed at 12 seconds (60 second blocks) and the next trial started immediately after the previous trial (see Figure 1).

### Analysis: Assessment of Psychostimulant Sensitization

On each session measures of both subjective drug effects and peripheral physiological processes were obtained. The measurement and analysis of these data have been presented in detail elsewhere [70]. Here, we provide a brief summary of these results for completeness.

Subjective drug effects were assessed using the Addiction Research Centre Inventory (ARCI) for amphetamine [72]–[74], the Profile of Mood States (POMS) [75], and Visual Analogue Mood Scales [76] at baseline and every 60 minutes for 240 minutes. Subjects were asked hourly to score each item for “how they feel at the present moment”. Physiological data (eye-blink rate, pulse and blood pressure, BP) were also collected (seated, following a resting period of 5 min). Eye-blink rate was taken as the average number of blinks over a 3 minute period at rest.

We anticipated behavioural (subjective) sensitization to amphetamine to mirror previous findings [62]–[65], including enhanced amphetamine-like experience, amphetamine-induced euphoria (ARCI-MBG), profile of mood states activity-vigour, alertness and attentiveness and positive affect as well as sensitization of resting eye-blink rate [70]. These hypotheses were tested using a Group × Administration/Session repeated measures analysis of variance (rmANOVA) for each dependent variable, using a level of significance of p<0.05 with Greenhouse-Geisser correction. All calculations were performed using SPSS15 for Windows.

### Analysis: Reward Gambling Task

A repeated-measures ANOVA (Group-by-Session-by-Trial-type (i.e. reward or control)-by-probability (of winning)) was used to test for between-group differences in reaction time. To confirm a relationship between any significant behavioural and subjective sensitization observed, we tested for correlations between both sensitised measures after correcting for individual differences in inter-session plasma amphetamine concentration (partial correlation). All calculations were performed using SPSS 15 for Windows.

### Analysis of Functional MRI Data

After pre-processing, including realignment, image distortion correction [77], [78], and normalisation, statistical analysis was carried out using the general linear model (GLM) [79], [80] as implemented in Statistical Parametric Mapping 2 (SPM2; Wellcome Trust Centre for Neuroimaging, London, UK). Each subject’s EPI data were normalised to a MNI EPI template. Two 1^st^ level (single subject) GLMs were employed. For analysis of task-related activations in general we constructed a model that represented gambling and control trials separately but did not distinguish task phases and used parametric modulation (second order polynomial expansion of the probability of winning, i.e. wheel-type). For analysing the main effects of interest, i.e. phase- and probability-specific activations, we constructed separate regressors encoding the task phases (Decision, Anticipation, Wins, and Losses) for each of the 3 wheel-types (i.e. probability of winning) for both the gambling and control conditions. In all cases, the vectors encoding the onset and duration of trials were convolved with a canonical hemodynamic response function [81]. Both models also included six regressors encoding volume to volume movement as nuisance regressors. The data were high-pass filtered (cut-off 128s) and corrected for serial correlations using a first-order autoregressive model.

At the group level, we first employed a repeated measures Session × Task Phase × Probability ANOVA model in the placebo group and explored the main effects of task, and probability. We then employed three Group × Session × Probability repeated-measures ANOVAs to test for Group × Session interactions, the appropriate test for sensitization-related effects, and Group × Session × Probability effects during rewarded decision-making, reward anticipation, and reward receipt (wins> losses). Statistical Parametric Maps (SPMs) of the t-statistic were constructed adjusting the maximum likelihood estimators for non-sphericity using restricted maximum likelihood. For both F-tests and t-tests, SPMs were thresholded at p<0.05 following family-wise error (FWE) correction for multiple testing in anatomically predefined volumes of interest (see below). To assess the significance of activations outside a priori regions of interest, we corrected for multiple comparisons (FWE) across the whole brain.

In regions where we had a priori hypotheses regarding the effects of repeated amphetamine exposure we corrected for multiple comparisons across the joint volume of these regions. In all cases, independently-derived (i.e. anatomically predefined) ROIs were employed to prevent biased statistical analyses [82]. For the striatum, we employed masks for the subdivisions of the striatum as defined anatomically by Mawlawi et al., respectively [83]. We defined the midbrain ROI as a sphere (10mm radius) around the peak coordinate in the SN/VTA reported by Wittmann et al. [84]. Additionally, for the orbitofrontal cortex and amygdala, bilateral anatomical masks were generated using the Automated Anatomical Labelling atlas [85] as implemented in Wake Forest University (WFU) PickAtlas. These ROIs, albeit unilateral, were used to extract regional parameter estimates for correlation analysis with behavioural measures of sensitization for the striatal subdivisions, midbrain and amygdalae. Although group plasma concentration of amphetamine did not differ between sessions, there was considerable variability in amphetamine concentration in the blood plasma within-subject and thus partial correlation analysis, which controlled for session-to-session difference in amphetamine plasma concentration, was employed.

### Subjective and Behavioural Sensitization to the Effects of Amphetamine

The analysis of subjective and behavioural sensitization effects is reported in detail elsewhere [70]. In brief, we found evidence for sensitization of subjective effects as demonstrated by significant Group-by-Session interactions for amphetamine-like experience (p = 0.015), drug-induced euphoria (p<0.009), Activity-Vigour scale (p = 0.018) and Dreamy-Attentive scale (p = 0.019). In contrast, physiological measurements (pulse, eye-blinks, and blood pressure) did not show evidence of sensitization.

### Decision-Making Reaction Time

As expected, we found evidence that reaction times were significantly influenced by trial-type (i.e. rewarded or control; F_(1,20)_ = 30.72, *p*<0.001) and probability of winning (F_(2,40)_ (2,40) = 10.23, *p*<0.001), with faster reaction times in during reward trials and trials with a higher-probability of winning. However, unexpectedly, we also observed a significant main effect of session (F_(1,20)_ = 49.72, *p*<0.001) suggestive of a practice effect, despite the fact that participants were trained on the task prior to scanning. We found no evidence for a Group × Session (*p*<0.569) or Group × Session × Trial Type (*p*<0.974) interactions.

### Task-Related BOLD Responses

**Figure 2:**
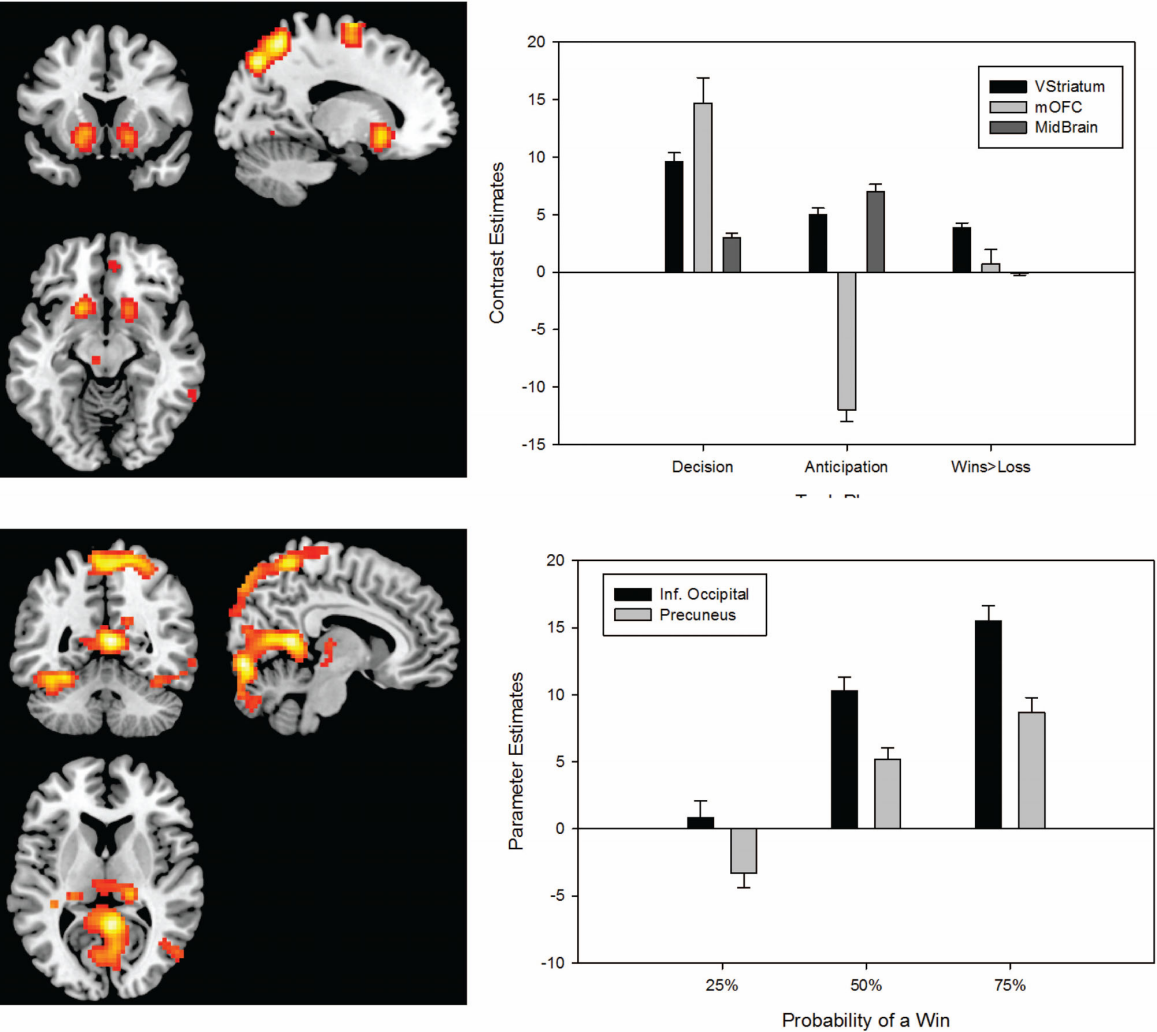
Upper Panel: Brain regions identified as displaying sensitivity to the task phases (i.e. decision, anticipation, wins and losses) in the placebo group (left panel). Parameter estimates for key dopaminergic and reward-related areas showing a significant main effect of task (right panel). Lower Panel: Brain regions where BOLD signal was modulated by reward probability the placebo group (left panel). Parameter estimates from the occipital cortex and precuneus, regions that display a significant main effect of reward probability. All parameter estimates reflect the mean response in arbitrary BOLD units. Results are shown with the standard error of the mean.

In accord with previous results from similar tasks [68], [69] we found a main effect of task phase in the placebo group in a large scale fronto-parietal network, including the cingulate and insular cortices, thalamus and basal ganglia. Furthermore, in the placebo group we also observed a main effect of probability in the dorso-medial parietal and occipital lobes (Figure 2 and Table S1). Finally, while we found no phase-by-probability interactions which were significant following whole-brain correction for multiple comparisons. However, using an *a priori* ROI we found evidence of a significant task-phase by probability interaction in the right limbic striatum which survived small volume correction (Z-score 3.25; p = 0.038; [21 15 −3]).

### Decision-making

**Figure 3:**
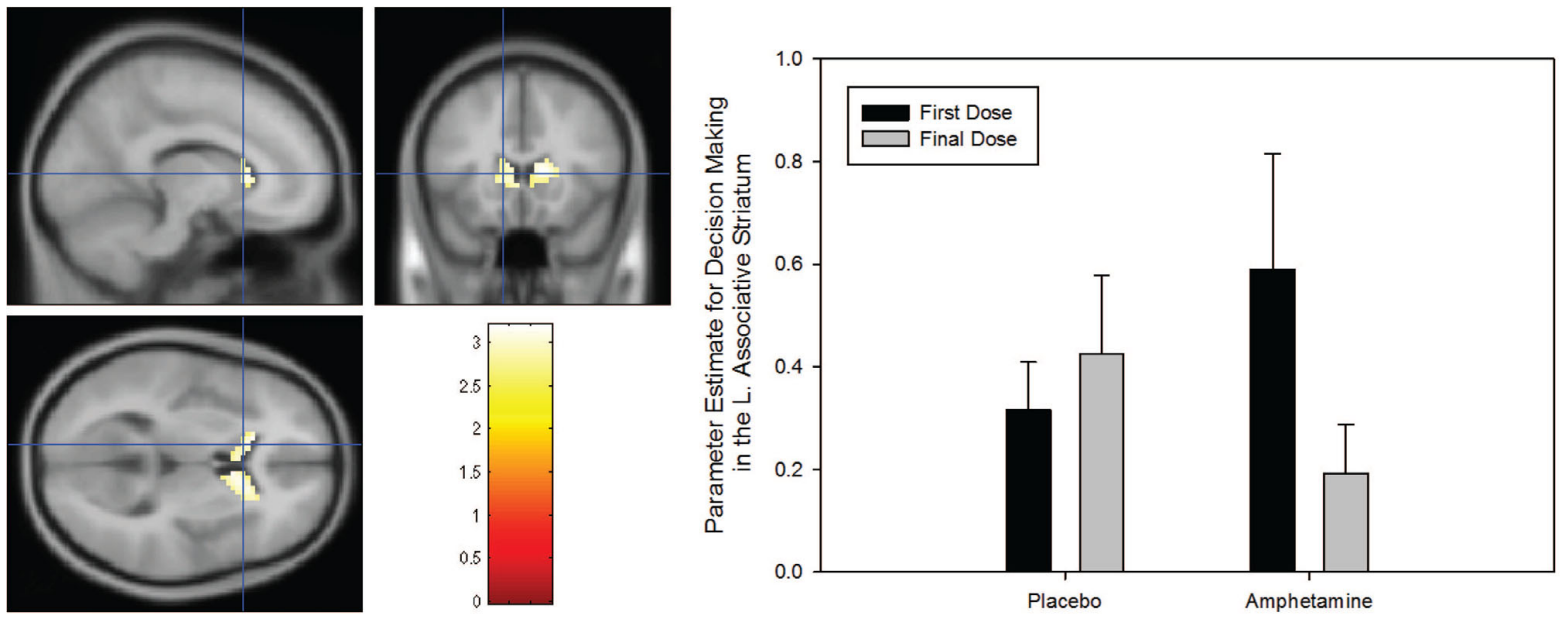
Significant Group × Session interaction in the caudate nucleus during the decision-making phase of our gambling task (p<0.05, corrected). The right panel shows parameter estimates (in the order placebo at scan 1, placebo at scan 2, AS at scan 1 (before sensitisation), AS at scan 2 (after sensitisation)) from the mean from an associative striatal ROI. Note that this plot is merely used to illustrate the nature of the interaction effect. All parameter estimates reflect the mean response in arbitrary BOLD units. Results are shown with the standard error of the mean.

We found evidence for a significant Group × Session interaction in decision-making related BOLD responses in the left ([−6 12 12], Z-score = 3.13, *p-corrected* = 0.038) and right caudate nucleus ([12 0 21], Z-score = 3.04, *p-corrected* = 0.05). As is clearly shown in figure 3, this interaction is driven by a significant reduction in decision-making related BOLD signal following sensitization. However, we found no evidence for a significant Group × Session × Probability interaction during decision-making.

### Anticipation

**Figure 4:**
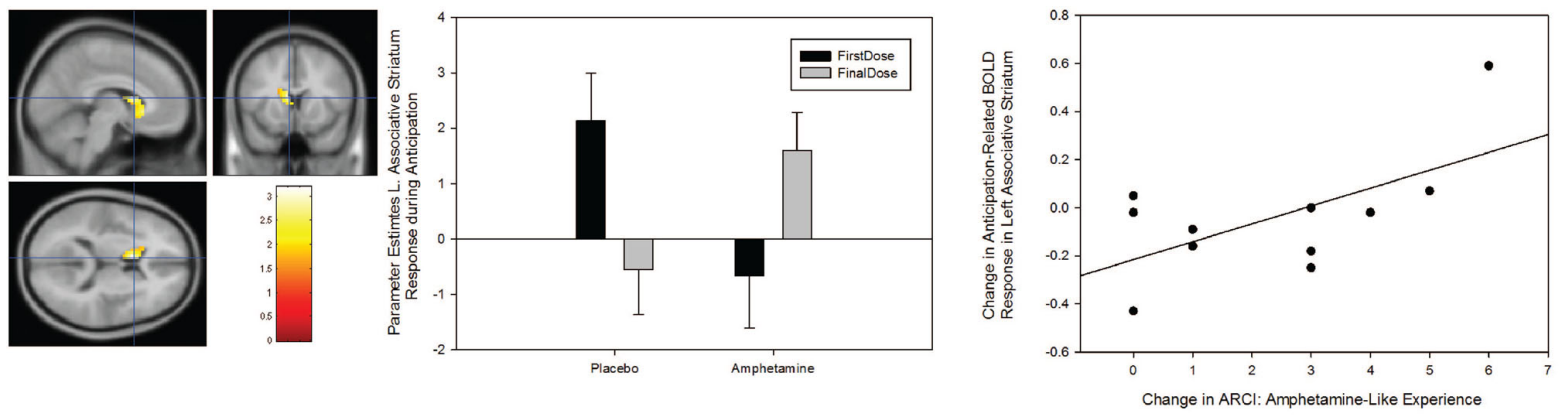
Significant Group × Session interaction (p<0.05, corrected) during the anticipation phase of our gambling task (left panel). To illustrate the nature of the interaction effect, the middle panel shows parameter estimates (in the order placebo at scan 1, placebo at scan 2, AS at scan 1 (before sensitisation), AS at scan 2 (after sensitisation) from the mean response within an associative striatal ROI. The graph on the right shows the correlation between sensitisation-related change in striatal BOLD signal during anticipation and the change in subjective response to amphetamine. All parameter estimates reflect the mean response in arbitrary BOLD units. Results are shown with the standard error of the mean.

As during the decision phase, we also found evidence for a significant Group × Session interaction in anticipation-related BOLD response in the left ([−15 24 6], Z-score = 3.15, *p-corrected* = 0.043) and right caudate nucleus ([9 12 9], Z-score = 3.14, *p-corrected* = 0.038). However, unlike the effect during decision making, and shown in figure 4, this interaction is driven by a significant increase in anticipation-related BOLD signal in the amphetamine group. To demonstrate that this effect was related to sensitization, we used a partial-correlation analysis (controlling for individual between-session differences in plasma amphetamine concentration) to test for a significant relationship between the change in anticipation-related BOLD signal in the caudate nucleus and sensitization of subjective measures of amphetamine-like experience. This analysis found that sensitization to amphetamine’s subjective effects (ARCI: Amphetamine) was positively correlated with the change in BOLD response during reward anticipation – over and above anticipation of a non-rewarded outcome – in the right caudate nucleus (r = 0.623, *p*_(1-tailed)_ = 0.027). Again, we found no evidence for a significant Group × Session × Probability interaction.

### Outcome Processing (Wins>Loss in Rewarded Trials)

**Figure 5:**
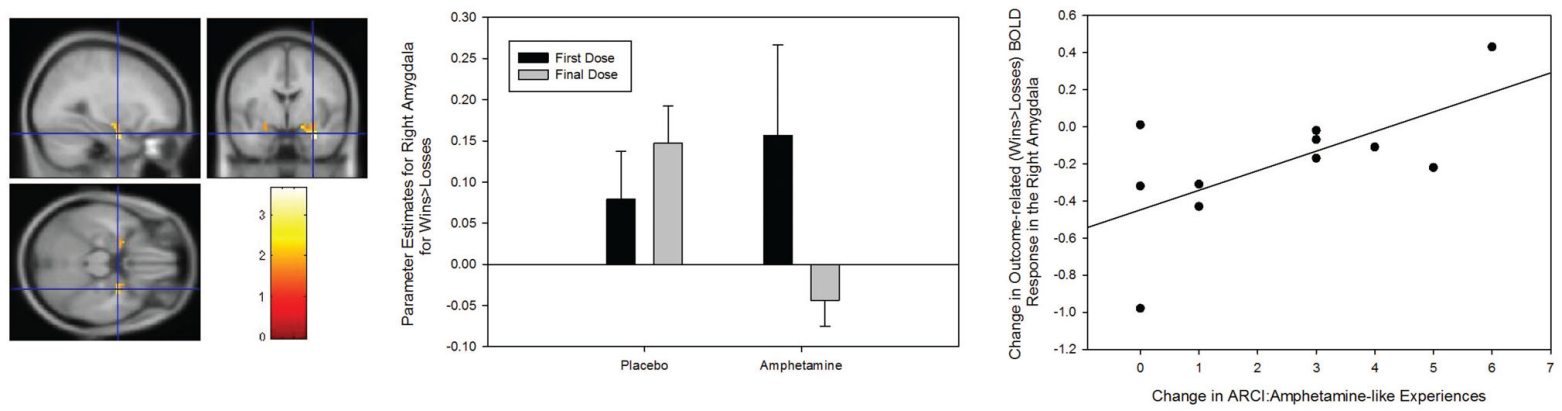
Significant Group × Session interaction (p<0.05, corrected) in the right amygdala during the outcome phase (i.e. Wins>Loss) of our gambling task (left panel). To illustrate the nature of the interaction effect, the middle panel shows parameter estimates (in the order placebo at scan 1, placebo at scan 2, AS at scan 1 (before sensitisation)), AS at scan 2 (after sensitisation) from peak voxel within the right amygdala. Additionally, the graph on the right shows the correlation between sensitisation-related change in amygdala BOLD signal during outcome-processing and the change in subjective report of amphetamine-like experience. All parameter estimates reflect the mean response in arbitrary BOLD units. Results are shown with the standard error of the mean.

We found evidence for a Group × Session interaction in outcome-related BOLD response in the amygdalae bilaterally. However, this interaction was driven by a significant reduction in amygdala BOLD signal change in response to wins compared to losses following repeated amphetamine-exposure effect in the right amygdala ([33 −3 −27], Z-score = 3.53, *p-corrected* = 0.006), (see Figure 5). Again, partial-correlation analysis (controlling for individual between-session differences in plasma amphetamine concentration) was used to test for a significant relationship between the change in outcome-related BOLD signal in the amygdala and sensitization of subjective measures of amphetamine-like experience. This analysis found that sensitization to amphetamine’s subjective effects (ARCI: Amphetamine) was positively correlated with the change in BOLD response to rewarded outcomes compared to losses (r = 0.636, *p* = 0.048).

## Discussion

This study found enhanced subjective responsiveness to amphetamine consistent with earlier work on dopaminergic sensitization in humans [62]–[65], though in contrast, physiological sensitization effects (changes in blink rate or blood pressure) were not observed and there were no differential effects on any aspects of reaction time compared to those receiving placebos. The fMRI results suggests a significant sensitization effect in the caudate nucleus and amygdala. Following repeated amphetamine exposure, the caudate nucleus showed reduced BOLD signal during decision-making, but enhanced BOLD activity during reward anticipation, which was correlated with the degree of sensitization. The amygdala BOLD response to reward outcomes was reduced following repeated amphetamine exposure, and this change was correlated with the degree of sensitization sensitization.

Surprisingly, despite our previous finding of a significantly faster reaction time following sensitization during a working memory [70], we did not find any sensitization of response time here. This is puzzling, but it may reflect a floor effect with little additional improvement compared to performance following the first amphetamine administration. Alternatively, the significant session effect observed may have masked the effect. This session effect is unexpected because participants were pre-trained on the task in an attempt to minimise these effects. This is a potentially important confound of our analysis of the BOLD response during decision-making, although the modelling of these events as a fixed 2-second window may ameliorate some of its impact. Nonetheless, the results of the decision-making phase of this task must be considered with this potential confound in mind.

The observed changes in the “associative striatum” (caudate nucleus) are in accord with previous PET studies of sensitization in healthy humans [65], cue-induced DA release in addiction [28] and drug-induced DA release in schizophrenia [17], [18]. Sensitization is also associated with an accelerated development of behaviours which are mediated by dorsal striatal DA transmission, namely stereotypy [86], outcome-insensitive behaviour [52] and stimulus-response “habit” formation [87]–[89]. While these findings are consistent with enhanced DA release in the dorsal striatum – posited linked to increased driving of ascending striato-nigrostriatal loop circuitry [90], [91] by sensitised mesolimbic stimulation of accumbens D_1_ receptors – the opposing direction of the effects is surprising. DA release blunts spontaneous neuronal activity in the neostriatum and accumbens and increases the efficacy of glutamatergic signalling at dendritic spines [92]. It is likely that this reflects an interaction between differential task-related cortico-striatal inputs (e.g. hippocampus or prefrontal cortex), task-evoked DA release and elevated basal dopamine concentrations following sensitization, indeed, these factors likely explain the lack of a sensitization-related change in the BOLD contrast (rewarded vs non-rewarded) conditions in the ventral (limbic) striatum. Specifically regarding the associative striatum, the reduced response observed during decision-making may reflect the blunting of the normal response in this region due to elevated synaptic dopamine [93]. Importantly, changes in the placebo group also contributed to this interaction, and likely reflect a change in the confidence regarding the reward delivery, given the change in reaction time discussed above. Furthermore, in the amphetamine group this effect was not significantly related to the degree of sensitization seen when individual differences were examined, and therefore may reflect a more general impact of repeated amphetamine exposure. Together, these findings, including a potential behavioural confound (i.e. a change in reaction time) suggest we should show some caution regarding the observed interaction during rewarded decision making.

We also observed a significant interaction in the associative (ventro-medial) caudate nucleus during reward anticipation. Importantly, as there are no motor responses during this condition and thus the changes observed are not confounded by changes in reaction time. Nonetheless, the interaction was driven both by a reduced responsiveness is the placebo group and increased anticipation-related activity, during rewarded trials compared to those when no reward was available. Importantly, elevated anticipation-related activity was significantly correlated with the degree of sensitization. We propose that this effect is driven by elevated excitability of striato-nigrostriatal loops [94] due to excessive nucleus accumbens dopamine release, with a resultant aberrant recruitment of the dorsal caudate during reward anticipation. This mechanism may be linked to the faster development of response habits following sensitization in rodents [89], a mechanism also implicated in addiction [95] and is linked to “dopamine-dependent serial connectivity between the ventral and dorsal striatum” [96]. These theories are also consistent with the incentive sensitization model [97], and our findings likely support the idea that sensitization alters the motivational significance of environmental events and cues in humans.

Amygdala dysfunction is argued to be a core pathophysiological mechanism in the development of addiction [98], [99] and has been demonstrated to be disrupted in schizophrenia [100]. Whilst commonly associated with the processing of fearful stimuli, there is a considerable body of evidence suggesting that the amygdala is also recruited during reward learning and Pavlovian behavioural responses [101], [102] and is seen in neuroimaging studies of reward outcome sensitivity – that is gains over losses [103]. The amygdala is heavily targeted by mesolimbic DA neurons which strongly modulate its activity [104]. It has been implicated in reward-seeking behaviour [105] and can drive cue-dependent drug-seeking behaviour [106], [107]. Concerning sensitization, the ability of the basolateral amygdala to modulate medial prefrontal neurons is augmented following a single acute amphetamine exposure but blunted by repeated amphetamine exposure, a process which depends on mesolimbic DA signalling [108]. Overall, it is possible that the reduced sensitivity to differential outcomes (i.e. gains> loss) may reflect a sustained elevation of amygdala activity associated with kindling, a process related to sensitization or perhaps more likely, the effects of an elevation in mesolimbic dopamine in this region. The apparently paradoxical positive correlation between sensitization to the drug’s subjective effects and the change in the response (i.e. reduction) of the amygdala, but given that fMRI is non quantitative and dependent upon BOLD contrast, this result could reflect a change either in reward sensitivity (i.e. to wins) or perhaps more likely, an increased responsiveness of the amygdala to losses. These findings speak potentially to two separate neuroplastic mechanisms at play. Specifically, at a group level the amygdala may display an enhanced sensitivity to losses, perhaps consistent proposals of allostatic changes in opponent processes following drug withdrawal-related to negative emotional states [109], which it is proposed my drive reinstatement of drug use. However, those individuals who have a greater propensity to develop sensitization, are to some degree protected from this effect. Importantly, these subjects are necessarily dysphoric at the time of scanning, in fact the visual analogue scales would suggest otherwise, but they had consumed amphetamine shortly before the scan. Additionally, while the neuroplastic (potentially allostatic) effects may endure, the subjective effects of withdrawal may not last long after so few exposures to such a low dose. This finding may be of particular importance given the amygdala’s role in updating value representations and attribution of incentive salience to environmental cues, such as the reward wheel, which remained on screen during outcome delivery.

Contrary to our expectations, sensitization was not associated with changes in either the nucleus accumbens or in the orbitofrontal cortex (OFC) during reward outcome processing. In the nucleus accumbens, this might be explained by saturation of post-synaptic D_1_ receptors (a ceiling effect), given the strong relationship between D_1_ stimulation and accumbens BOLD signal [110]. Concerning the lack of sensitization effects in the OFC, our acquisition deliberately employed a long repetition time to permit us to collect a large number of thin slices to minimise the susceptibility “drop-out” effects [111]. This was chosen because the OFC is vulnerable to drop out effects in fMRI and changes in the orbitofrontal cortex were predicted. The lack of OFC sensitization effects is all the more puzzling given the observed changes in the amygdala, a brain region with strong reciprocal connectivity with the OFC [112], [113]. An examination of the imaging masks confirmed that the absence of an effect was not related to a lack of coverage which accords with observed task-related recruitment of this region. It could be argued that this absence simply reflects the fact that our participants have only received 4 doses of amphetamine, a far shorter dosing regimen than those used in rodent and primates studies. However, there is some evidence that hyper-excitability of orbitofrontal neurons is one of the earliest observed neuroadaptations in rodents [58]. As described elsewhere [70], 10/11 subjects in each group were homozygous for the Val158Met polymorphism. However, these same subjects were also genotyped for a novel polymorphism of the DAT gene which has been linked to the propensity to abuse cocaine [114]. We found a relatively even split of the risk allele in these subjects, and in a brief report published elsewhere [115], we found that this polymorphism significantly modulated the effects of acute amphetamine on reward-related recruitment of orbitofrontal cortex. The effect of this polymorphism on the development of sensitization remains to be determined, but it may explain our failure to detect a significant sensitization-related change in the OFC.

There are a number of limitations with this study that should be highlighted. Firstly, the sample size is relatively small. Nonetheless, we have previously demonstrated an effect of sensitization during working memory [70] and motor sequence learning [71]. Furthermore, the evidence that sensitization of mesolimbic dopamine release is evident up to one year after first expression [65] was published during our data collection and raised concerns about collecting a larger sample. Secondly, the repetition time for the scan was quite long, which perhaps reduced our sensitivity to detect some effects. The fact that we used a fixed dose of 20mg for all participants, rather than a weight titrated dose was agreed with our ethics committee and local pharmacy on the basis of a typical dose used clinically. Clearly, titrated doses would have been preferable, and may explain some of the heterogeneity in the observed effects. Note however, that there was no evidence for a significant difference in drug plasma levels during the scanning on the first and last visit and therefore, while some variability on the expression of sensitization was anticipated, it is possible that some component of this could be explained by this fixed dose. While we screened all participants for recent drug use on every visit, our information on previous drug history was based solely on subjective report. However, the level of previous drug use was extremely low, particularly in our amphetamine group. While, some very infrequent recreational use was reported by some participants, none reported prolonged administration of therapeutic stimulants for either weight-loss or treatment of ADHD, which would have been an exclusion criterion. Furthermore, participants’ doctors were contacted before they were finally recruited to ensure that pre-existing conditions that would bar entry to the study were not concealed. As our study was focused on sensitization as a model of dopamine dysregulation in schizophrenia, rather than addiction, we did not collect an index of drug liking vs drug wanting. This was an unfortunate oversight, as given the incentive sensitization model which has be suggested, in the aberrant salience hypothesis [51] to be important in schizophrenia. In an ideal world, the participant could have been dosed and waited in the scanning environment prior to the scanning beginning. Unfortunately, this was not possible, but the context was carefully controlled with all participants staying in the same room for all 4 sessions, with dosing and scanning at the same fixed times of the day. We feel that this minimised the potential impact of any contextual confounders. Finally, while all participants were of normal healthy weight when recruited, and none reported any change in their eating habits, it is possible that some of the observed effects could be driven by changes in body weight because we did not weigh participants on every visit.

We found evidence for blunted responses in the caudate and amygdala, suggestive of altered processing within salience and motivational circuits during decision-making and reward processing in the amphetamine group, although these effects were likely reflecting more general effects of repeated amphetamine exposure. However, the enhanced dorsal striatal responses during reward anticipation are suggestive of findings in rodents and may speak to increased motivational drive for reward, and processes which would ultimately result in reduced sensitivity to reward outcomes, such as is seen in drug addiction and patients with schizophrenia. Overall, this data speaks to disruption of neural systems and processes linked to RPE-dependent learning mechanisms, but perhaps not in a sensitization specific manner. Whereas, sensitization-related effects were evident related to the anticipation of a rewarded, compared to a non-rewarded, outcome. Amphetamine sensitization in otherwise healthy volunteers implicates many of the same structures and processes observed previously in rodents, an suggests that this translational and translatable model yields insights to potentially important mechanisms underlying the development of both addiction and schizophrenia, and may explain their relatively high comorbidity [12], [116].

## Supporting Information

Table S1 (Top) Cluster information, (cluster size, coordinates, statistics and labels) for brain regions displaying a significant main effect of Task Phase in the placebo group. (Bottom) Cluster information, (cluster size, coordinates, statistics and labels) for brain regions displaying a significant main effect of Probability in the placebo group.
(DOCX)
