# Correction: Amphetamine Sensitization Alters Reward Processing in the Human Striatum and Amygdala

**DOI:** 10.1371/journal.pone.0218478

**Published:** 2019-06-12

**Authors:** Owen G. O’Daly, Daniel Joyce, Derek K. Tracy, Adnan Azim, Klaas E. Stephan, Robin M. Murray, Sukhwinder S. Shergill

The following information is missing from the Funding statement: This study was supported by the European Research Council Executive Agency (ERC).
