# Supplementary material for: Amphetamine Sensitization Alters Reward Processing in the Human Striatum and Amygdala
Source: PLoS One. 2014 Apr 9;9(4):e93955. doi: 10.1371/journal.pone.0093955 (PMC3981726; doi:10.1371/journal.pone.0093955)
Supplement: Table S1 — (Top) Cluster information, (cluster size, coordinates, statistics and labels) for brain regions displaying a significant main effect of Task Phase in the placebo group. (Bottom) Cluster information, (cluster size, coordinates, statistics and labels) for brain regions displaying a significant main effect of Probability in the placebo group. (DOCX) [file pone.0093955.s001.docx]

| Placebo: Task Phase |  |  |  |  |  |
| --- | --- | --- | --- | --- | --- |
| Region Label | Size | Equiv Z | x(mm) | y(mm) | z(mm) |
| Precuneus | 1223 | Inf | -12 | -60 | 63 |
| Precuneus |  | Inf | 12 | -57 | 57 |
| Sup. Parietal |  | Inf | -27 | -54 | 60 |
| Sup.Frontal | 391 | Inf | -21 | -6 | 66 |
| Sup. Frontal | 308 | Inf | 27 | 0 | 57 |
| Sup.Frontal |  | 6.99 | 18 | -6 | 72 |
| Putamen | 152 | 6.9 | 18 | 15 | -9 |
| Mid. Cingulate | 128 | 6.72 | -3 | -39 | 33 |
| Putamen | 108 | 6.37 | -15 | 12 | -9 |
| Angular | 107 | 6.14 | -42 | -69 | 45 |
| Sup.Frontal | 107 | 5.82 | -15 | 36 | 51 |
| Sup. Medial Frontal |  | 5.39 | -3 | 45 | 54 |
| Sup. Medial Frontal |  | 5.07 | -6 | 54 | 45 |
| Mid. Temporal | 103 | 5.78 | -63 | -39 | -6 |
| Mid.Cingulate | 10 | 5.58 | -12 | -24 | 39 |
| Lingual | 10 | 5.38 | 9 | -24 | -12 |
| Calcarine | 25 | 5.37 | -9 | -48 | 3 |
| Insula | 44 | 5.32 | 39 | 24 | 3 |
| Inf. Frontal |  | 5.12 | 48 | 18 | 3 |
| Thalamus | 23 | 5.11 | 6 | -15 | 9 |
| SMA | 12 | 5.05 | 0 | -3 | 63 |
| Angular | 17 | 5.03 | 48 | -66 | 45 |
| Lingual | 17 | 4.95 | -9 | -81 | -9 |
| Medial Orbitofrontal | 20 | 4.88 | -6 | 45 | -15 |
| Middle Occipital | 7 | 4.87 | 42 | -69 | 3 |
| Sup. Frontal | 8 | 4.84 | -21 | 21 | 54 |
| Inf. Parietal | 10 | 4.83 | -51 | -30 | 39 |
| Middle Occipital | 7 | 4.82 | -45 | -72 | 3 |
| Insula | 2 | 4.69 | -30 | 21 | 6 |
| Calcarine | 1 | 4.67 | 21 | -60 | 18 |
| Medial Orbitofrontal | 1 | 4.66 | 12 | 42 | -15 |
| Lingual | 1 | 4.59 | 18 | -63 | -6 |
|  |  |  |  |  |  |
| Placebo: Probability |  |  |  |  |  |
| Region Label | Size | Equiv Z | x(mm) | y(mm) | z(mm) |
| Inf. Occipital | 33 | 5.19 | -51 | -66 | -12 |
| Precuneus | 27 | 5.19 | -3 | -51 | 9 |
| Calcarine | 27 | 5.19 | -3 | -84 | -6 |
| Precuneus | 4 | 4.65 | -3 | -51 | 63 |
